# Genome-Wide Association Study for Somatic Skeletal Traits in Duroc × (Landrace × Yorkshire) Pigs

**DOI:** 10.3390/ani14010037

**Published:** 2023-12-21

**Authors:** Xin Gao, Shenping Zhou, Zhihong Liu, Donglin Ruan, Jie Wu, Jianping Quan, Enqin Zheng, Jie Yang, Gengyuan Cai, Zhenfang Wu, Ming Yang

**Affiliations:** 1 College of Animal Science and Technology, Zhongkai University of Agriculture and Engineering, Guangzhou 510225, China; gaoxin_21@outlook.com (X.G.); shenpingzhou1109@163.com (S.Z.); 13060843790@163.com (Z.L.); 2 College of Animal Science and National Engineering Research Center for Breeding Swine Industry, South China Agricultural University, Guangzhou 510642, China

**Keywords:** DLY pigs, somatic skeletal traits, GWAS

## Abstract

**Simple Summary:**

In China, soup is widely perceived as a nutritional tonic, and pig bones are a primary ingredient in its preparation. Consequently, the market price of pig bones, even when of equal weight, surpasses that of pork, particularly in southern China. Despite this economic significance, the trait of pig bone weight has not been a focus in pig breeding programs due to the inherent challenges associated with in vivo measurement. The genetic selection method stands out as an effective approach for targeting the trait of pig bone weight in pig breeding. Recognizing its significance, our research endeavors have delved into molecular genetics to gain insights into the genetic aspects of pig bone weight. In order to unravel the genetic underpinnings of specific traits, we employed bioinformatics methodologies to conduct an analysis on three-way hybrid commercial pigs. Our primary objective was to identify genetic markers and genes associated with four distinct bone weight traits. Utilizing genome-wide association studies (GWAS), we scrutinized a population comprising 571 three-way hybrid commercial pigs to pinpoint single nucleotide polymorphisms (SNPs) and genes linked to these traits. The results identified twelve genes, namely *OPRM1*, *SLC44A5*, *WASHC4*, *NOPCHAP1*, *RHOT1*, *GLP1R*, *TGFB3*, *PLCB1*, *TLR4*, *KCNJ2*, *ABCA6*, and *ABCA9*, that were related to four bone weight traits. This comprehensive genetic insight provides valuable information on the mechanisms governing bone growth and weight in the context of pig breeding.

**Abstract:**

The pig bone weight trait holds significant economic importance in southern China. To expedite the selection of the pig bone weight trait in pig breeding, we conducted molecular genetic research on these specific traits. These traits encompass the bone weight of the scapula (SW), front leg bone weight (including humerus and ulna) (FLBW), hind leg bone weight (including femur and tibia) (HLBW), and spine bone weight (SBW). Up until now, the genetic structure related to these traits has not been thoroughly explored, primarily due to challenges associated with obtaining the phenotype data. In this study, we utilized genome-wide association studies (GWAS) to discern single nucleotide polymorphisms (SNPs) and genes associated with four bone weight traits within a population comprising 571 Duroc × (Landrace × Yorkshire) hybrid pigs (DLY). In the analyses, we employed a mixed linear model, and for the correction of multiple tests, both the false discovery rate and Bonferroni methods were utilized. Following functional annotation, candidate genes were identified based on their proximity to the candidate sites and their association with the bone weight traits. This study represents the inaugural application of GWAS for the identification of SNPs associated with individual bone weight in DLY pigs. Our analysis unveiled 26 SNPs and identified 12 promising candidate genes (*OPRM1*, *SLC44A5*, *WASHC4*, *NOPCHAP1*, *RHOT1*, *GLP1R*, *TGFB3*, *PLCB1*, *TLR4*, *KCNJ2*, *ABCA6*, and *ABCA9*) associated with the four bone weight traits. Furthermore, our findings on the genetic mechanisms influencing pig bone weight offer valuable insights as a reference for the genetic enhancement of pig bone traits.

## 1. Introduction

Pigs are among the most extensively consumed animals globally, and the pricing of their various parts varies based on factors such as quality and region. In recent years, pig by-products, including pig bones, have gained increasing popularity in markets throughout China, resulting in a steady escalation of prices over the years [1]. For instance, in the southern region of China, pork bones such as spareribs and leg bones command higher prices than most cuts of pork. Bone weight, being a trait associated with body shape, serves as a reliable predictor of height and weight [2]. Previous studies have identified multiple candidate genes associated with body weight and body conformation traits in Duroc × (Landrace × Yorkshire) hybrid pigs (DLY) [3]. Additionally, in recent years, breeders have been leveraging swine body measurement traits for selective breeding purposes [4,5,6]. Therefore, it is crucial to comprehend the genetic foundations of key frame bone weight traits in pigs, encompassing scapula weight (SW), front leg bone weight (including humerus and ulna) (FLBW), hind leg bone weight (including femur and tibia) (HLBW), and spine bone weight (SBW). These discoveries may offer novel perspectives on pig bone development and lay the groundwork for employing molecular breeding techniques to enhance bone weight [7,8].

In recent years, advancements in sequencing technologies have enabled the acquisition of high-density single nucleotide polymorphisms (SNPs). Leveraging this genomic tool, researchers can conduct genome-wide association studies (GWAS) in both humans [9] and pigs [10,11,12] to pinpoint genetic variants linked to polygenic traits. Despite the identification of a total of 844,800 quantitative trait loci (QTLs) associated with complex traits in the pig QTL database, only six QTLs have been specifically associated with pig bone weight characteristics [13] (accessed on 23 June 2023). Su et al. [14] employed the linear model least squares method to identify five bone weight quantitative trait loci (QTLs) on SSC7, reaching the level of significance at the chromosome level, based on microsatellite marker genotypes. Similarly, in 2007, Zhang et al. [15] utilized the same method to discover a significant QTL for bone weight on chromosome 6. Despite the identification of these six QTLs over a decade ago, progress in the genetic improvement of pig bone weight has been limited.

Currently, GWAS studies on pig bone traits have predominantly concentrated on parameters such as bone density and length, crucial for assessing limb robustness. For example, Jiang et al. [16] identified a highly significant association with bone mineral density (BMD) around 41.7 Mb on SSC6. Similarly, Mao et al. [17] conducted whole-genome scanning using 183 microsatellite markers across the pig genome, revealing 35 QTL for limb bone lengths and 2 for femoral BMD. However, research specifically focused on pig body bone weight remains limited. This limitation may be attributed to the necessity for post-slaughter measurements of bone weight in pigs, which imposes considerable financial and human resource demands. 

Our study aims to identify SNPs and potential candidate genes linked to pig bone weight traits. To achieve this goal, we conducted a GWAS employing a porcine 50K SNP gene chip in a cohort of 571 Duroc × (Landrace × Yorkshire) hybrid pigs. The aim was to identify significant SNPs and potential candidate genes associated with SW, FLBW, HLBW, and SBW traits. It is noteworthy that our work marks the first GWAS study on these bone weight traits in pigs.

## 2. Materials and Methods

### 2.1. Ethics Statement

All animal handling procedures were conducted under the guidelines for the care and use of laboratory animals of the Ministry of Agriculture of the People’s Republic of China. The study has been approved by the Animal Care Committee of Zhongkai University of Agriculture and Engineering (ZUAE) (Guangzhou, China). 

### 2.2. Animals and Phenotype

This study used 571 DLY pigs bred by Wens Food Group Co., Ltd. (Guangzhou, China), produced by 31 Duroc boars and 128 Landrace × Yorkshire sows. When the pigs reached 180 days of age, they were transported to China Resources Wufeng Meat Investment Co., Ltd. (Shenzhen, China) for slaughter. Half of the carcass was segmented, and the phenotypic value was recorded. After skinning, scalding, scrapping, and eviscerating, the scapula, front leg bone, hind leg bone, and spine bones of 571 pigs were sampled by four experimenters, and four weight traits (SW, FLBW, HLBW, and SBW) were measured [18]. 

### 2.3. Genotyping and Quality Control

Genotyping was processed as described by Ding et al. [19]. Genomic DNA was extracted from pig ear tissue by phenol-chloroform extraction, and DNA quality was evaluated by light absorption ratio (A_260/280_ and A_260/230_) and electrophoresis. For genotyping, the GeneSeek Porcine 50K SNP BeadChip (Neogen, Lincoln, NE, USA) was used for the 571 DLY pigs, which contained 50,643 SNPs. After genotyping, quality control (QC) was conducted using PLINK v1.09 [20]. We ruled out the individuals with a call rate of <95%; SNPs with a call rate < 95%, minor allele frequency (MAF) <0.01, and Hardy–Weinberg equilibrium *P* < 1 × 10^−6^ were deleted. Moreover, QC also involved the removal of SNPs with no position information or located on the sex chromosomes. After QC, a set of 34,821 effective SNPs from all DLY pigs were used for GWAS. 

### 2.4. Population Structure Analysis

Principal component analysis (PCA) divides individuals into different subgroups according to genetic differences and analyzes the genetic structure of populations. We performed PCA of the SNP dataset using the software GCTA v1.94.0beta [21]. This analysis helped adjust and minimize the confounding effect of population structure in GWAS [22]. Furthermore, quantile-quantile (Q-Q) plots were also constructed in R 4.3.1 to evaluate the impact of potential population stratification on GWAS after adding the top five principal components in the GWAS model. 

### 2.5. Genome-Wide Association Analyses

A matrix of genomic similarity index between individuals within the population was utilized for single-trait GWAS using GEMMA v0.98.5 [23], a typical GWAS analysis software based on a mixed linear model (MLM). With a univariate linear mixed model, GWAS was performed independently on the SW, FLBW, HLBW, and SBW traits. The association analyses were carried out using the following MLM model:

(1) y=Wα+Xβ+u+ε

where y is the trait phenotype value; W is the fixed effect matrix, including the top five eigenvectors of PCA, sex, slaughter batch, and live weight; α is the corresponding coefficient including intercept; X is the SNP genotype; β indicates the effect size of the marker; u is a random effect, *u*~*MVN_n_* (*0*,*λτ*^−1^*K*); ε represents residuals, *ε*~*MVN_n_* (*0*,*τ*^−1^*I_n_*); *λ* is the ratio between the two variance components; *τ*^−1^ is the residual variance; *K* is the standardized correlation matrix estimated by GEMMA v0.98.5; *I_n_* is the unit matrix; and *n* is the number of animals. 

The genome-wide suggestive thresholds were *P* < 1/*N*, where *N* is the number of SNPs. To deal with the Bonferroni correction’s overly conservative false negative results, we also set a more relaxed threshold, that is, the false discovery rate (FDR) [24]. An FDR of 0.01 was used as the threshold value for identifying SNPs suggestively associated with each trait [25]. The threshold *P*-value was defined as follows:

(2) $$P=FDR\times \frac{N}{M}$$

where N represents the number of SNPs in the GEMMA-based GWAS results with *P* < 0.01, ordered in ascending order according to their effects. M represents the number of SNPs that surpassed the threshold value. In addition, haplotype block analysis was performed on chromosome regions with multiple significant SNPs using PLINK v1.09 and Haploview v4.2 software [26]. The default parameters of Haploview 4.2 [26] (MAF > 0.05, Mendelian error < 2, and *P*-value < 10^−3^ for the HWE test) were used to define the linkage disequilibrium (LD) blocks of the SNPs.

### 2.6. Estimation of Genetic Parameters and the Explained Phenotypic Variance

GCTA v1.94.0beta was used to estimate the genetic correlation between any two of the four traits by performing a bivariate genome-based restricted maximum likelihood (GREML) analysis [21,27]. The formula for calculating the genetic correlation coefficient is as follows:

(3) $$r_{g}=\frac{\sigma_{g1g2}}{\sigma_{g1}\sigma_{g2}}$$

where $r_{g}$ is the genetic correlation coefficient between two traits; the subscripts “1” and “2” represent the two traits; $\sigma_{g1g2}$ refers to the genetic covariance; and $\sigma_{g}$ represents the square root of the genetic variance for the trait (captured by all SNPs).

The restricted maximum likelihood method was used to estimate the phenotypic variance explained by the significant SNPs for four bone weight traits using GCTA v1.94.0beta [28]. The phenotypic variance explained by the significant SNPs was calculated with the following model:

(4) $$y=X\beta +g+\varepsilon \;\mathrm{with}\;var\left(y\right)=A_{g}\sigma_{g}^{2}+I\sigma_{\varepsilon }^{2}$$

where y refers to the vector of phenotype value; β is a vector of fixed effects; X is an incidence matrix for β; g represents the vector of the aggregate effects of all the qualified SNPs for the pigs within one population; I is the identity matrix; $A_{g}$ is the GRM; $\sigma_{g}^{2}$ corresponds to the additive genetic variance captured by either the genome-wide SNPs or the selected SNPs; and $\sigma_{\varepsilon }^{2}$ refers to the residual variance. 

### 2.7. Candidate Genes and Functional Annotation

Candidate genes nearest the significant SNPs were obtained based on the *Sus scrofa* 11.1 genome version database (https://asia.ensembl.org/index.html, accessed on 10 December 2022). According to the published literature and GO and KEGG analysis of candidate genes [29,30], the biological processes of the genes associated with the signals were determined [31,32,33,34]. Kyoto Encyclopedia of Genes and Genomes (KEGG) and Gene Ontology (GO) analyses were performed with all candidate genes using KOBAS 3.0 (http://bioinfo.org/kobas/, accessed on 11 September 2023) [35,36]. Fisher’s exact test was used to evaluate the significance of the enrichment items using *P* < 0.05 as the standard [37]. The aim is to explore genes involved in pathways and biological processes.

## 3. Results

### 3.1. Phenotype Statistics and Heritability Estimation

The values of phenotypic statistics and heritability (h^2^) are shown in Table 1. A total of four groups of high-quality phenotypic data were obtained from 571 pigs, including 507 SW, 506 FLBW, 542 HLBW, and 430 SBW. In this study, the average SW, FLBW, HLBW, and SBW in DLY pigs were 0.63 kg, 0.97 kg, 1.72 kg, and 1.45 kg, respectively. Notably, the average weight of the hind leg and spine bones is heavier than the other two bones. All four traits exhibited high coefficients of variation ranging from 11.10% to 15.75%, indicating a high genetic variation in bone mass and great potential for breeding improvement. SBW displays the lowest estimated heritability of 0.279, while the other bone weights had high estimated heritability ranging from 0.470 to 0.672. 

The genetic and phenotypic correlations of the four traits are shown in Table 2. The results showed that there were moderate to high positive genetic correlations between the four traits. Apart from a moderate genetic correlation between HLBW and SBW (r = 0.33), there is a high genetic correlation between the other traits (r > = 0.50), implying that these traits can be improved simultaneously in one breeding scheme. The correlation between the weight traits of the front leg bone and the hind leg bone was 0.78, while the correlation between the scapula, the front leg bone, and the hind leg bone is moderate.

### 3.2. Population Structure Analysis

**Figure 1:**
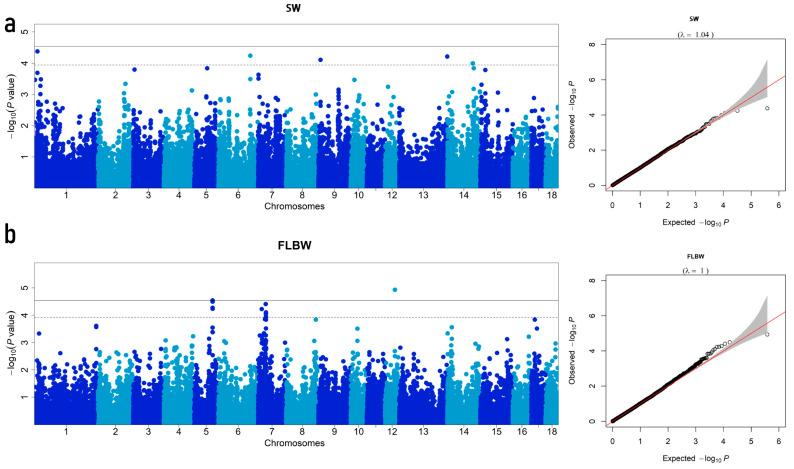

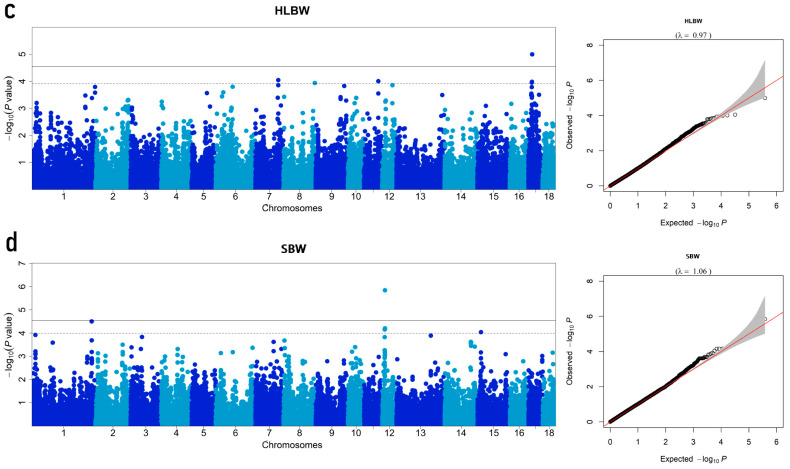
Manhattan and Q–Q plots of genome-wide association studies for SW, FLBW, HLBW, and SBW in the DLY population. (**a**) SW: scapula weight; (**b**) FLBW: front leg bone weight; (**c**) HLBW: hind leg bones weight; (**d**) SBW: spine bones weight. The Manhattan plots of the GWAS are plotted with the x-axis representing the chromosomes, the y-axis representing the −log10 (*P*-value), and different colors indicating various chromosomes. In the Manhattan diagram, the real line and the dotted line represent the whole-chromosome (suggestive) Bonferroni correction threshold and the FDR threshold, respectively. The x-axis represents the actual measured value of −log10 (*P*-value) and the y-axis represents the observed value of −log10 (*P*-value) in the Q-Q plots, and λ represents the coefficient of expansion.

It is widely recognized that population stratification can increase the false positive rate of GWAS results. We performed PCA on the SNP dataset to minimize false positives using GCTA v1.94.0beta [21]. We then selected the top five eigenvectors as covariates in the association analysis model to correct the population structure. Additionally, the Manhattan and Q-Q plots of the four analyzed traits are shown in Figure 1. The figure’s expansion coefficient (λ) is between 0.97 and 1.06, suggesting that no evident population stratification exists in the experimental population studied.

### 3.3. Summary of GWAS Results

Manhattan plots of GWAS for the four traits and corresponding Q-Q plots are shown in Figure 1. Through the Bonferroni threshold (*P* < 2.86 × 10^−5^), only four SNPs were detected to be associated with bone weight traits. Furthermore, a total of 26 associated SNPs were detected under relaxed FDR thresholds of *P* < 1.15 × 10^−4^ (SW), *P* < 1.19 × 10^−4^ (FLBW), *P* < 1.23 × 10^−4^ (HLBW), and *P* < 1.02 × 10^−4^ (SBW). SNPs that were found to be significantly associated with SW, FLBW, HLBW, and SBW for the DLY population are listed in Table 3. In brief, a total of 26 SNPs were significantly associated with bone weight traits, including five SNPs related to SW, ten SNPs related to FLBW, six SNPs related to HLBW, and five SNPs associated with SBW.

According to Figure 1a and Table 3, we detected five SNPs significantly associated with SW, located on chromosomes 1, 6, 9, 13, and 14. These five SNPs exceeded the threshold *P* < 1.15 × 10^−4^; however, no SNP exceeded the Bonferroni threshold. Figure 1a shows an expansion coefficient (λ) of 1.04. Among these significant SNPs, MARC0065727, the most significant one, is located within the *OPRM1* gene on SSC1 and explains 3.92% of the phenotypic variation. 

For FLBW, ten significant SNPs were identified on SSC5, SSC7, and SSC12 with a λ of 1 (Figure 1b and Table 3). These ten SNPs all exceeded the FDR threshold of *P* < 1.19 × 10^−4^. Four of these significant SNPs were located on SSC5 with an interval of 131 kb. Both DIAS0000686 and DRGA0006075 are located within the *WASHC4* gene, with only a 16 kb distance between them. Furthermore, located on the gene *NOPCHAP1*, the distance between ASGA0026453 and ALGA0033115 is 17 kb, explaining 4.99% and 2.09% of phenotypic variation, respectively. However, the most significant SNP named WU_10.2_12_44505563 only explained 1.54% of the phenotypic variation and is located within the *RHOT1* on chromosome 12. Additionally, both H3GA0020988 and ALGA0040570 are located on the *GLP1R* gene, with a distance of 36 kb. 

Six SNPs were significantly associated with HLBW, with a λ of 0.97. All six SNPs exceeded the genome-wide significance threshold of *P* < 1.23 × 10^−4^ (Figure 1c and Table 3). The top SNP, DRGA0016598, is located 487 kb downstream of the *PLCB1* gene and accounted for 3.50% of the phenotypic variance. In addition, two significant SNPs were located within a 2 Mb distance with DRGA0016598 on SSC17.

For SBW, five significant SNPs were identified located on SSC1, SSC12, and SSC15, with a λ of 1.06 (Figure 1d and Table 3). All these SNPs exceeded the FDR threshold of *P* < 1.02 × 10^−4^. The most significant SNP, WU_10.2_12_11424336, is located on the *ABCA6* gene and explains 5.59% phenotypic variation. Moreover, the SNP named WU_10.2_12_11794440 is 56 kb downstream of the top SNP, located on the *ABCA9* gene.

### 3.4. Candidate Genes and Function Analysis

A total of 23 candidate genes were identified, which either overlapped or were located near the significant SNPs (Table 3). To gain insight into the genes and pathways involved in the biology of somatic skeletal traits, we performed KEGG and GO analysis of these 23 candidate genes across all four traits using KOBAS 3.0 [35,36]. Enriched terms with the criteria of *P* < 0.05 were selected to further explore the enriched pathways and biological processes. Interestingly, when FDR = 0.05, the candidate genes were significantly enriched to several KEGG pathways and GO terms (Table 4), including pathways related to bone growth and metabolism, such as rheumatoid arthritis, inward rectifier potassium channel activity, and negative regulation of nitric oxide biosynthetic process, among others. 

## 4. Discussion

**Figure 2:**
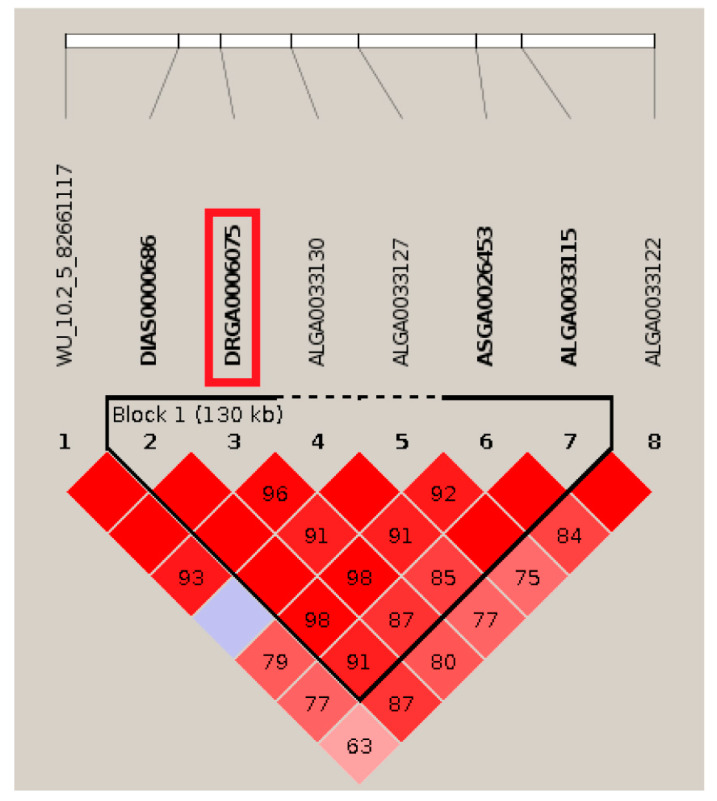
Haplotype blocks for significant SNPs within black triangular wireframe indicate a haplotype block composed of significant SNPs located on SSC5 for FLBW in DLY pigs. A haplotype block of 130 kb contains four significant SNPs (DIAS0000686, DRGA0006075, ASGA0026453, and ALGA0033115), including the top SNP DRGA0006075 within the red wireframe.

Pig bones contain a variety of trace elements and proteins, and they have a significantly higher energy content than pork. In some regions of China, pig bones are even more popular than pork. However, there has been limited exploration into the genetic mechanisms underlying bone weight in pigs. For instance, the pig QTL database has only identified six QTLs related to bone weight [13]. Measuring bone weight in live pigs is challenging, as most assessments are post-slaughter. In our current study, we conducted the first-ever GWAS analysis for SW, FLBW, HLBW, and SBW in DLY pigs. This analysis uncovered 26 SNPs associated with bone weight traits among 571 DLY pigs using the FDR threshold. However, few significant SNPs were identified when using the more stringent Bonferroni threshold. Chen et al. demonstrated that using a relaxed threshold in a larger sample can yield a better true positive rate than in a smaller sample [38]. Armstrong has noted that many experts do not advocate for the application of Bonferroni correction to a series of goodness-of-fit tests. This is because reducing the likelihood of type I errors increases the likelihood of type II errors, thereby elevating the possibility of falsely fitting models in certain datasets [39]. In Armstrong’s perspective, many studies have applied conservative corrections uncritically, potentially leading to the oversight of “real” effects. Therefore, our study employed both conservative and relaxed correction methods for multiple hypothesis testing to provide a more comprehensive analysis, with the results from both methods complementing each other. The estimated heritabilities for SW, FLBW, HLBW, and SBW fall within the range of 0.28 to 0.67, indicating moderate to high heritability. While bone weight is a complex quantitative trait influenced by numerous genes, its genetic background remains relatively unexplored. Notably, our study is one of the largest GWAS endeavors for bone weight to date. Remarkably, 16 significant SNPs collectively explained over 2% of the phenotypic variance, with five surpassing 5%. This suggests that these markers hold potential for genomic selection in the context of pig bone weight. Furthermore, the QTLs discovered in this study were not previously associated with bone weight (see Figure 2). 

A noteworthy SNP, MARC0065727, is located within *OPRM1* (opioid receptor mu 1), the gene coding for the μ opioid receptor. Speculation has arisen regarding the influence of genetic factors on bone mineral density (BMD), and *OPRM1* has been linked to BMD in Caucasian women, particularly at the hip, spine, and body [40]. BMD acts as an indirect indicator reflecting bone mass and fracture risk. Additionally, the SNP WU_10.2_7_105204658, located within *TGFB3* (transforming growth factor beta 3) on chromosome 7, has shown a strong association with trabecular bone mineral density (vBMD) in the human femoral neck [41]. This is particularly significant for assessing bone strength and the risk of osteoporosis. Therefore, *OPRM1* and *TGFB3* are potential candidate genes for influencing bone weight. Another significant SNP, ASGA0029572, is situated within the *SLC44A5* gene. Previous research has demonstrated that *SLC44A5* (solute carrier family 44 member 5) regulates bovine birth weight. Sugimoto et al. discovered that calves with lower *SLC44A5* expression exhibited larger sizes due to increased cell proliferation [42]. Their study further revealed that knockdown of *SLC44A5* reduced choline release, while increased *SLC44A5* expression had the opposite effect. Given that choline is a key component of cell cytomembranes, this suggests that *SLC44A5* plays a crucial role in regulating bone cell proliferation. The SNP WU_10.2_12_44505563 is also located within *RHOT1* (Ras homolog gene family, member T1). Previous analysis has observed the upregulation of *RHOT1* genes in pagetic osteoclasts [43], implying that *RHOT1* could be a potential candidate gene for FLBW. 

In this study, we have identified significant associations between specific SNPs and FLBW. Two SNPs form a region that houses one known gene on SSC7, *GLP1R* (glucagon-like peptide 1 receptor). *GLP1R* is a G-protein-coupled receptor for *GLP-1*, which, when activated, regulates insulin secretion and thus influences osteoporosis [44]. Interestingly, Zeng et al. reported that *GLP1R*, as a target gene, suppresses AMPK signal activation under the influence of MiR-27a-3p. This, in turn, inhibits pre-osteoblast differentiation and autophagy, ultimately impacting bone formation [45]. Since osteoblasts and osteoclasts work in tandem to maintain bone homeostasis and disruptions can lead to osteoporosis, the *GLP1R* gene emerges as a strong candidate for FLBW due to its influence on bone growth. Moreover, DIAS0000686 and DRGA0006075 explained 12.19% of the phenotypic variation, and both were located on a protein coding gene *WASHC4* (WASH complex subunit 4). Courtland et al. [46] found that genetic damage of *WASHC4* affects mice’s cognitive function and causes obvious progressive dyskinesia in mice. Meanwhile, there were also two significant SNPs, ASGA0026453 and ALGA0033115, which explained 7.08% of the phenotypic variation, located on the *NOPCHAP1* (NOP protein chaperone 1) gene, which is also a protein-coding gene. However, the correlation between these two genes and the bone weight of the front legs still requires further exploration.

Consumers often prefer back-leg bones due to their ability to hold a considerable amount of bone marrow. In the case of HLBW, the gene *TGFB3* not only impacts BMD but also influences the entire life cycle of chondrocytes, mediating various cellular responses, including cell survival, proliferation, migration, and differentiation [47]. Therefore, *TGFB3*’s regulatory effect on cartilage development is particularly crucial. Moreover, SNP DRGA0016598 is close to *PLCB1* (phospholipase C beta 1), a gene that regulates pig growth traits. Meng et al. have demonstrated that *PLCB1* is involved in the gonadotropin signaling pathway, indicating its contribution to pig growth during puberty and its consequential effect on pig bone weight [48]. 

For SBW, the top SNP, WU_10.2_12_11424336, is within *ABCA6* (ATP-binding cassette subfamily A member 6). This gene has been linked to bone health and osteoporosis in humans and animals [49]. Similarly, another gene, *KCNJ2*, is located near the MARC0086052 SNP and has been reported to regulate bone formation and mineralization in women [50]. Hence, *ABCA6* and *KCNJ2* emerge as strong candidate genes for influencing SBW. Another noteworthy SNP, INRA0007356, is located near the *TLR4* (toll-like receptor 4) gene, associated with bone density and skeletal health in humans and mice [51]. Consequently, this SNP and the *TLR4* gene may be vital in regulating pig bone weight. Lastly, one SNP (WU_10.2_12_11794440) is located on chromosome 12 within the *ACBA9* (ATP-binding cassette subfamily A member 9) gene, which has not previously been implicated in bone health. Further investigation is required to ascertain the functional significance of this association. 

Functional annotation has revealed several pathways and biological processes within the 23 positional candidate genes associated with bone weight traits. Most of the significant KEGG pathways and GO terms are related to bone development processes, such as rheumatoid arthritis, inward rectifier potassium channel activity, and negative regulation of nitric oxide biosynthetic process (Table 4). For HLBW and SBW, *TGFB3* and *TLR4* were enriched to the pathway of rheumatoid arthritis. Rheumatoid arthritis is a disease characterized by bone loss [52]. Since severe bone loss can lead to a decrease in bone weight, it is conceivable that the candidate genes regulate bone weight. For SBW, *KCNJ2* was enriched to inward rectifier potassium channel activity. Increased potassium intake significantly reduces renal calcium excretion, which favors bone health [53,54]. These results indicate that potassium ion content influences the SBW trait in pigs. The *OPRM1* was significantly enriched with negative nitric oxide biosynthetic process regulation for SW. Jin et al. [55] demonstrated that nitric oxide modulates bone anabolism by regulating osteoblast glycolysis and differentiation. Given the intimate connection between bone anabolism and overall bone growth and development, it is conceivable that these genes also play a role in determining bone weight.

Our findings provide valuable insights into the genetic basis of bone weight in pigs and may inform future breeding strategies aimed at improving pig bone health. However, further studies are warranted to confirm our findings and delve into the underlying mechanisms behind these genetic associations in regulating bone mass.

## 5. Conclusions

Our study has identified 26 SNPs and 12 promising candidate genes that exhibit significant associations with bone weight traits in DLY pigs. Our results provide new insights into the genetic architecture of somatic skeletal traits in pigs. Moreover, some significant SNPs associated with somatic skeletal traits in this study may be helpful for marker-assisted selection in pig breeding.

## Figures and Tables

**Table 1:** Descriptive statistics and heritability for SW, FLBW, HLBW, and SBW traits in the Duroc × (Landrace × Yorkshire) hybrid pigs (DLY) population.

Trait	N	Mean (±SD)	Min/Kg	Max/Kg	C.V./% ^1^	*h^2^* (±SE)
SW	507	0.63 ± 0.08	0.39	0.87	12.71	0.470 ± 0.077
FLBW	506	0.97 ± 0.11	0.67	1.35	11.79	0.672 ± 0.073
HLBW	542	1.72 ± 0.19	1.1	2.32	11.10	0.629 ± 0.071
SBW	430	1.45 ± 0.23	0.85	2.46	15.75	0.279 ± 0.088

^1^ Coefficient of variation (C.V.).

**Table 2:** Phenotypic and genetic correlations among the four bone traits in the DLY population.

	SW	FLBW	HLBW	SBW
SW	1	0.41	0.40	0.13
FLBW	0.77 + 0.11	1	0.78	0.21
HLBW	0.52 + 0.15	0.86 + 0.06	1	0.26
SBW	0.86 + 0.34	0.50 + 0.31	0.33 + 0.30	1

Phenotypic correlations (above the diagonal) and genetic correlations (below the diagonal) among SW, FLBW, HLBW, and SBW traits within the DLY population. All of the phenotypic correlation coefficients are significant, with *P*-value < 0.05.

**Table 3:** Significant SNPs and associated genes for the four bone traits in the single-trait association analysis.

Trait	SSC ^1^	SNP ID ^2^	Position (bp) ^3^	MAF ^4^	*P*-Value	PVE/% ^5^	Candidate Gene	Distance (bp) ^6^
**SW**	1	MARC0065727	12,629,436	0.474	4.20 × 10^−5^	3.92	*OPRM1*	within
	6	ASGA0029572	137,682,936	0.26	5.74 × 10^−5^	3.72	*SLC44A5*	within
	9	ALGA0108358	9,320,080	0.218	7.80 × 10^−5^	2.55	*NEU3*	within
	13	WU_10.2_13_217373583	207,442,473	0.32	6.12 × 10^−5^	1.52	*UBE2G2*	−2059
	14	ASGA0091963	109,404,872	0.452	0.000101038	0.55	*CRTAC1*	14,180
**FLBW**	5	DIAS0000686	79,444,610	0.302	5.31 × 10^−5^	5.79	*WASHC4*	within
	5	**DRGA0006075**	79,461,126	0.298	2.85 × 10^−5^	6.40	*WASHC4*	within
	5	ASGA0026453	79,557,821	0.256	3.18 × 10^−5^	4.09	*NOPCHAP1*	within
	5	ALGA0033115	79,575,118	0.277	5.81 × 10^−5^	2.99	*NOPCHAP1*	within
	7	H3GA0020119	16,266,396	0.328	5.93 × 10^−5^	1.45	*CDKAL1*	within
	7	MARC0074546	33,359,087	0.166	3.89 × 10^−5^	1.47	*ZFAND3*	−19,127
	7	ALGA0040524	34,151,758	0.209	9.01 × 10^−5^	1.69	*ENSSSCG00000027778*	within
	7	H3GA0020988	34,502,131	0.267	0.000113219	1.10	*GLP1R*	within
	7	ALGA0040570	34,538,678	0.267	7.84 × 10^−5^	1.61	*GLP1R*	4049
	12	**WU_10.2_12_44505563**	42,721,240	0.303	1.17 × 10^−5^	1.54	*RHOT1*	within
**HLBW**	7	WU_10.2_7_105204658	99,159,898	0.26	9.05 × 10^−5^	1.04	*TGFB3*	within
	8	WU_10.2_8_144898378	135,537,530	0.363	0.000115895	2.40	*SCD5*	within
	11	H3GA0032120	62,244,494	0.28	9.89 × 10^−5^	1.34	*GPC5*	98,256
	17	ALGA0093437	15,401,211	0.302	0.000111254	4.48	*ENSSSCG00000043546*	69,056
	17	WU_10.2_17_17981232	16,253,154	0.315	0.000104166	7.44	*ENSSSCG00000025527*	224,053
	17	**DRGA0016598**	17,258,153	0.269	1.01 × 10^−5^	3.50	*PLCB1*	487,159
**SBW**	1	INRA0007356	257,807,729	0.312	3.16 × 10^−5^	4.27	*TLR4*	−236,881
	12	MARC0086052	10,012,238	0.365	7.02 × 10^−5^	3.19	*KCNJ2*	−337,171
	12	**WU_10.2_12_11424336**	11,240,882	0.247	1.44 × 10^−6^	5.59	*ABCA6*	within
	12	WU_10.2_12_11794440	11,296,596	0.265	6.30 × 10^−5^	5.07	*ABCA9*	within
	15	ALGA0084013	14,781,096	0.108	9.16 × 10^−5^	2.48	*ENSSSCG00000042807*	94,194

^1^ *Sus scrofa* chromosome; ^2^ the bold data indicate that the SNP exceeds the Bonferroni threshold (*P* < 2.86 × 10^−5^); ^3^ SNP position in Ensembl; ^4^ minor allelic frequency; ^5^ proportion of total phenotypic variation explained by each SNP; ^6^ the location of SNP upstream/downstream of the nearest gene.

**Table 4:** Significant KEGG PATHWAY and GO terms with four bone weight traits (*P* < 0.05).

Term	Database	ID	Gene Names	Corrected *P*-Value
Rheumatoid arthritis	KEGG PATHWAY	ssc05323	*TGFB3*, *TLR4*	0.032405
Inward rectifier potassium channel activity	Gene Ontology	GO:0005242	*KCNJ2*	0.003538
Negative regulation of nitric oxide biosynthetic process	Gene Ontology	GO:0045019	*OPRM1*	0.033125

## Data Availability

Data are contained within the article.
